# Impact of salinity on the morpho-biochemical traits of hydroponically cultivated *Spinacia oleracea* L.

**DOI:** 10.5114/bta/202318

**Published:** 2025-03-31

**Authors:** Archana A. Naik, Shekhar D. Tidke, Mahadev R. Chambhare, Ravindra D. Bansode, Panchshila S. Kabnoorkar

**Affiliations:** 1 Department of Botany, Savitribai Phule Pune University, Pune, Maharashtra, India; 2 Department of Biotechnology, Dr. D. Y. Patil Arts, Commerce and Science College, Pune, Maharashtra, India; 3 Department of Botany, SSM’s, Amruteshwar Arts, Commerce, and Science College, Pune, Maharashtra, India; 4 Vidya Pratishthan’s College of Agricultural Biotechnology, Pune, Maharashtra, India; 5 Department of Botany, Arts, Science and Commerce College, Pune, Maharashtra, India

**Keywords:** SDS-PAGE, *Spinacia oleracea*, hydroponics, salt stress, antioxidant assay

## Abstract

**Background:**

*Spinacia oleracea*, a nutrient-dense vegetable composed of 91% water, 4% starch, and 3% protein, is a staple in the Indian diet. However, salinity stress can hinder its growth. This study examines the effects of salinity on the morpho-biochemical traits of spinach in a hydroponic system.

**Materials and methods:**

Spinach seeds were sown on coco peat, and after 10 days, the seedlings were transferred to the hydroponic setup. The plants were treated with salt concentrations of ECe 0, 4.0, 6.0, 8.0, 10.0, and 12.0 ds/m, and various morpho-biochemical parameters were assessed at 10-day intervals.

**Results:**

In the control group, seed germination was 59.6 ± 1.45%, while increasing salt concentrations (4 to 10 dS/m) progressively reduced germination (50 ± 1.2% to 14 ± 2%). Root and shoot lengths (root: 14.16 ± 0.19 cm; shoot: 4.23 ± 0.28 cm at 4 dS/m), relative water content (47.5 ± 0.43% to 32.1 ± 0.86%), and leaf surface area (25.03 ± 0.26 to 9 ± 0.12 cm^2^) all declined with increasing salinity. Conversely, proline content (0.055 ± 0 to 0.12 ± 0 μg/ml) and antioxidant enzyme activity (SOD: 1.83±0.04/g protein; APX: 0.53 ± 0.1/mg protein; CAT: 0.0054 ± 0/mg protein) increased compared to the control. However, chlorophyll content (3.73 ±0.02 to 1.95 ± 0.03 mg/g at 12 dS/m) and protein content (0.13 ± 0 at 4 dS/m to 0.054 ± 0 μg/ml at 12 dS/m) decreased with rising salinity.

**Conclusions:**

Therefore, it is concluded that spinach grown hydroponically can tolerate salt stress up to ECe 6.0 dS/m after 30 days of treatments, and more increased (8.0 to 12 dS/m) salt concentration that adversely affects overall morpho-biochemical performance.

## Introduction

*Spinacia oleracea* is native to Central and Southwest Asia and is cultivated worldwide as an annual herb. It is a deciduous, early-maturing, cool-season crop grown for its green leaves and biological functions, including antimutagenic, antioxidant, and anticancer activities (Tewani et al., 2016; Wang et al., 2002). Between 2000 and 2017, Asia accounted for 94% of global spinach production, followed by Europe (3.1%), the Americas (2.2%), Africa (0.6%), and Oceania (0.1%). In 2016, worldwide spinach production reached 26.7 million metric tons (FAOSTAT, 2017).

In addition to vitamins A, C, K, and B (including thiamin, riboflavin, and niacin), raw spinach contains 3% protein, 4% starch, and essential minerals such as magnesium, sodium, phosphorus, manganese, calcium, potassium, iron, and folate, along with dietary fiber. The nutritional composition of Indian spinach per 100 g includes 3.2 g of protein, 4.3 g of carbohydrates, and 0.3 g of fat, with water comprising 91% of its total weight (Oladele and Olakunle, 2011).

Hydroponics is the technique of growing crops, vegetables, and other plants without soil in a liquid medium containing fortified nutrients (Heredia, 2013; Ranawade et al., 2017). The term hydroponics was coined by William Frederick Gericke, who also developed a commercial hydroponic system in the 1930s (Mugundhan et al., 2011). In contrast to soil gardening, hydroponic culture systems have been shown to have numerous benefits. They can be utilized with any size stream, are less prone to soil-borne diseases and parasites, and require very tiny space. Under identical conditions, hydroponically grown plants grow 30–50% faster than soil-grown plants (Haddad et al., 2009). Additionally, hydroponic farming enables crop cultivation in extreme environments, such as arid deserts and tundra (Heredia, 2013). Hydroponic-grown plant products with added trace elements for human health have the potential to reduce human diseases. According to research on selenium-enriched garlic, eating garlic cultivated in soilless cultures with an excess of selenium significantly lowers the risk of cancer (Ip et al., 1991). The hydroponic floating system has proven to be particularly effective for spinach cultivation in both ordinary conditions and in seawater (Caparrotta et al., 2019). Currently, plants require 17 essential elements for growth and development (Salisbury and Ross, 1994). One of the most widely used nutrient solutions for hydroponic plant cultivation is the Hoagland solution, first developed by Hoagland and Arnon in 1938 and later refined by Arnon in 1950 (Tavakkoli et al., 2010; Chambhare and Nikam, 2022). Stress is an environmental factor that limits plant productivity or leads to biomass loss (Grime, 1989; Karnwal, 2021). Abiotic stresses pose a significant challenge to the expansion of cultivated areas worldwide. Stress induced by high ionic concentrations reduces the water gradient, making it more difficult for water and nutrients to pass through the root membrane (Shelke et al., 2023; Mohamadzadeh et al., 2023). As a result, water uptake slows down and the osmotic implication spreads from the root membrane to the inner membrane, the ion concentration inside the plant changes the osmotic balance (Volkmar et al., 1998).

Salinity is a major stress factor affecting most vegetable and crop production (Shelke et al., 2019). Salinity-induced osmotic stress in the root zone primarily inhibits crop growth by disrupting physiological, biochemical, and molecular processes. Increasing salt concentrations visibly reduces plant growth, resulting in smaller leaves, dwarf stems, and, in some cases, fewer leaves (Dumicic et al., 2017). Salt stress affects nearly all physiological, biochemical, and molecular pathways in plants (Sardar et al., 2018; Shelke et al., 2023; Tidke et al., 2019). The equilibrium of C under salt-stressed conditions depends on exposure, and achieving positive values requires sufficient photosynthetic recovery (Dimitrova et al., 2017). Additionally, the low osmotic potential of saline solutions reduces water uptake, leading to physiological drought. This makes plants more sensitive to salinity, a key factor contributing to agricultural productivity, particularly in arid areas (Boyer 1982; Sonawane et al., 2022).

In this study, *S. oleracea* was grown hydroponically to investigate the effects of salt stress on plant growth. The study examined a wide range of morphological and biochemical properties. In this study, clay-free sandstone was used, and after 10 days, spinach seedlings were transferred from cocopeat to a hydroponic system. Plants were examined every 10 days for morphological and biochemical changes caused by the application of various salt concentrations. As a result, this study investigated a wide range of morphological and biochemical properties.

## Materials and methods

### Collection of certified seed material

Certified seed material of *S. oleracea* (spinach) variety “All Green” was procured from Naik Krushi Bhandar, Pune, Maharashtra, India. The seedlings were grown and maintained under hydroponic conditions in a greenhouse located in the botanical garden of the Department of Botany, Savitribai Phule Pune University, Pune, Maharashtra, India.

### Establishment of hydroponic cultures

**Figure 1:**
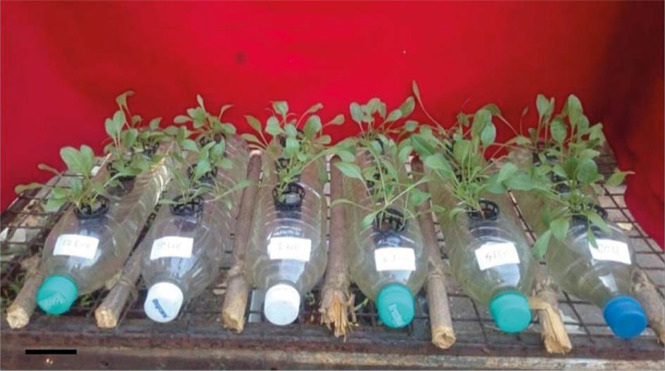
Hydroponically grown cultures of *Spinacia oleracea*

Table and plastic bottles were used to create the hydroponic arrangement as shown in Figure 1A hydroponic system was on a table, with sticks placed between two bottles to prevent interruption and put upright. To attach the net cup, a circular space was cut onto the surface of each bottle. The hydroponically grown spinach cultures were incubated at a temperature of 25 ± 2°C under an 8-h photoperiod (light intensity: 40 μmol m^-2^s^-1^) and 70% relative humidity.

### Seedling treatment and transfer on coco-peat

Spinach seeds were treated with a 0.1 M mercuric chloride (HgCl_2_) solution for 5 min, rinsed three times with double-distilled water, and sown in coco peat. After 10 days, the seedlings were transplanted into a hydroponic culture medium containing 0.2 mM ammonium nitrate (NH_4_NO_3_), 5.0 mM potassium nitrate (KNO_2_), 2.0 mM calcium nitrate (Ca(NO_3_)_2_), 2.0 mM magnesium sulfate (MgSO_2_), 0.1 mM monopotassium phosphate (KH_2_PO_4_), 0.05 mM NaFe(III) hydroxyethyl ethylenediamine triacetic acid (HEDTA), 0.01 mM boric acid (H_3_BO_3_), 0.005 mM manganese (II) chloride (MnCl_2_), 0.005 mM zinc sulfate (ZnSO_4_), 0.0005 mM copper (II) sulfate (CuSO_4_), and 0.0001 mM sodium molybdate (Na_2_MoO_3_) (Tavakkoli et al., 2010; Sneha et al., 2013). The nutrient solution was replaced every two days, and the pH was maintained between 5.6 and 6.0 (5.8 ± 0.2) using phosphoric acid and calcium carbonate. The electrochemical equivalent (ECe) of the hydroponic culture medium was adjusted to ECe 0.0, 4.0, 6.0, 8.0, 10.0, and 12.0 dS/m (deciSiemens per meter) using sodium chloride (NaCl).

### Morphological parameters

#### Seed germination percentage

After 10 days of sowing, the percent seed germination was calculated using the following formula (Brenchley and Probert, 1998).


$$\text{GP}=\frac{\text{NI}}{\text{N}}\times 100,$$

where *GP* – seed germination percentage, *Ni* – number of germinated seeds, and *N* – total number of seeds sown.

#### Root and shoot length measurement

The root and shoot lengths of hydroponically grown spinach seedlings were measured for all salt treatments after 10, 20, and 30 days. Measurements were taken using a scale and recorded in centimeters (cm).

#### Leaf relative water content

Leaf relative water content (LRWC) was determined using a modified method from Nikam et al. (2019). To minimize age-related variation, leaves were consistently collected from the mid-section of the plant. Individual leaves were removed from the stem and weighed to obtain the fresh weight. To determine the turgid mass, leaves were floated in distilled water inside a closed Petri dish. Leaf samples were weighed periodically after gently wiping off excess water with tissue paper. The samples were then placed in a preheated oven at 80°C for 48 h to obtain the dry weight. All mass measurements were recorded using an analytical scale and expressed in grams (g). The relative tissue water content was calculated using the formula (Yamasaki and Dillenburg, 1999):


$$\text{Leaf relative water content }(\text{LRWC \%})\text{ =}\frac{\left(\text{FW}-\text{DW}\right)}{\left(\text{TW}-\text{DW}\right)}\times 100,$$

where FW – fresh weight, DW – dry weight, TW – total weight.

#### Measurement of leaf area

To minimize age effects, leaves were collected from the mid-section of the plant. Individual leaves were removed, and leaf area was measured using graph paper, following a modified method by Pandey and Singh (2011). Outcome values are presented as square centimeters (cm^2^) area.

### Biochemical parameters

#### Determination of proline content

Proline content was determined following the method described by Bates et al. (1973). Leaf samples (0.5 g) from each treatment were homogenized in 3.0% (w/v) sulfosalicylic acid, and the homogenate was filtered through the Whatman No. 1 filter paper. The mixture was heated at 100°C for 1 h in a water bath after the addition of acidninhydrin and glacial acetic acid. The reaction was then stopped by placing the mixture in an ice bath. Proline was extracted with toluene, and the absorbance of the toluene fraction was recorded at a wavelength of 520 nm using a spectrophotometer. Proline concentration was determined using a calibration curve, and the proline content in control and NaCl-treated seedlings was quantified and expressed as μmol proline g^-1^ FW (Koca et al., 2007).

#### Protein estimation

Bovine Serum Albumin (BSA) is used as a standard against unknown concentrations of proteins estimated in the samples. A mixture of reagents recommended by the American biochemist Oliver Howe Lowry (Lowry et al., 1951) was added to the sample extracts and incubated for 15 min. Then, 0.5 ml of freshly prepared reagent 2 (1 part Folin Ciocalteau: 1 part water) was added to each sample, followed by 30 min of incubation in the dark. Absorbance was measured at 660 nm, and the protein content was expressed as mg BSA/g FW (Sarkar et al., 2020).

#### Chlorophyll estimation

Chlorophyll content in salt-treated and control spinach plants was estimated using the method of Arnon (1949). Fresh leaves (1.0 g) were finely cut and homogenized in 5.0 ml of 80% chilled acetone using a prechilled mortar and pestle in the dark. The homogenized extract was filtered through Whatman No. 1 filter paper and centrifuged at 10,000 rpm for 5 min. The supernatant was transferred to a separate vial, and the procedure was repeated until the residue became colorless. The absorbance of the final supernatant was measured at 645 and 663 nm against acetone as a blank using a spectrophotometer (Remi, C24). The chlorophyll a, chlorophyll b, and total chlorophyll contents were calculated using the following formulae:


$$\begin{array}{l} \text{Chlorophyll a }=12.7\;(\text{OD }663)-2.69\;(\text{OD }645)\times (\text{V}/1000)\times \text{W}\\ \text{Chlorophyll b }=22.9\;(\text{OD }645)-4.86\;(\text{OD }663)\times (\text{V}/1000)\times \text{W}\\ \text{Total Chlorophyll }(\text{mg}/\text{g})\;=20.2(\text{A}645)+8.02\;(\text{A}663)\times \frac{\text{V}}{1000\times \text{W}}, \end{array}$$

where *A* – absorbance, *OD* – optical density, *V* – volume of sample, *W* – weight of sample.

#### Antioxidant enzyme extraction and enzyme assays

Fresh leaf samples (200 mg) were homogenized in a chilled mortar and pestle with 2 ml of ice-cold 0.1 mM potassium phosphate buffer (pH 7.5) containing 1 mM EDTA (ethylenediamine tetraacetic acid), 1 mM PMSF (phenylmethylsulfonyl fluoride), and 5% (w/v) PVP (polyvinylpyrrolidone). The homogenates were centrifuged at 4°C for 20 min at 15,000 rpm, and the resulting supernatant was used as a crude extract for enzyme activity assays, including superoxide dismutase (SOD), catalase (CAT), and ascorbate peroxidase (APX) (Costa et al., 2002). SOD activity was determined by assessing its ability to inhibit the photochemical reduction of nitroblue tetrazolium. One unit of SOD activity was defined as the amount of enzyme required to achieve 50% inhibition of NBT reduction per minute at 560 nm (Dhindsa et al., 1981). CAT activity was measured by recording the decrease in absorbance at 240 nm for 3 min at 30-s intervals. The amount of decomposed hydrogen peroxide (H_2_O_2_) was calculated using its extinction coefficient (39.4 mM^–1^ cm^–1^), and enzyme activity was expressed as units per milligram of protein (Haida and Hakiman, 2018). APX activity was determined by monitoring the hydrogen peroxide-dependent oxidation of ascorbic acid, which was measured as a decrease in absorbance at 290 nm for 3 min at 30-s intervals. The amount of oxidized ascorbate was calculated using its molar extinction coefficient (2.8 mM^–1^ cm^–1^), and enzyme activity was expressed as units per milligram (mg) of protein (Haida and Hakiman, 2018).

#### SDS-PAGE analysis

The total protein content in spinach leaves was separated using polyacrylamide gel electrophoresis (SDS-PAGE) following the method described by Galyean and Laney (1980) and Tidke et al. (2019). Extracted protein aliquots (10 μg) were mixed with 2× loading buffer [0.09 M Tris-HCl (pH 6.8), 20% glycerol, 2% sodium dodecyl sulfate (SDS), 0.02% bromophenol blue, and 0.1 M dichloro-diphenyl-trichloroethane (DTT)], heated at 100°C for 5 min, and separated on a 12.5% polyacrylamide gel (1.5 mm thickness). The GelDoc Go System was used to capture images of protein bands.

#### Experimental design and statistical analyses

The experiments were arranged in a completely randomized design (CRD) with a minimum of 14 replicates per treatment and were conducted at least three times. Observations were recorded at specific time intervals and daily. Experimental data were analyzed using analysis of variance (ANOVA), and significant differences between means were determined using Duncan’s Multiple Range Test (DMRT) at a 5% significance level (*p* < 0.05) (Duncan, 1995). Data variations are expressed as mean ± standard error, and significant differences between means are indicated using different alphabets.

## Results and discussion

### Morphological parameters

Ten-day-old spinach seedlings were treated with different salt concentrations, and morphological parameters were examined at 10-day intervals.

#### Seed germination percentage (%)

**Figure 2:**
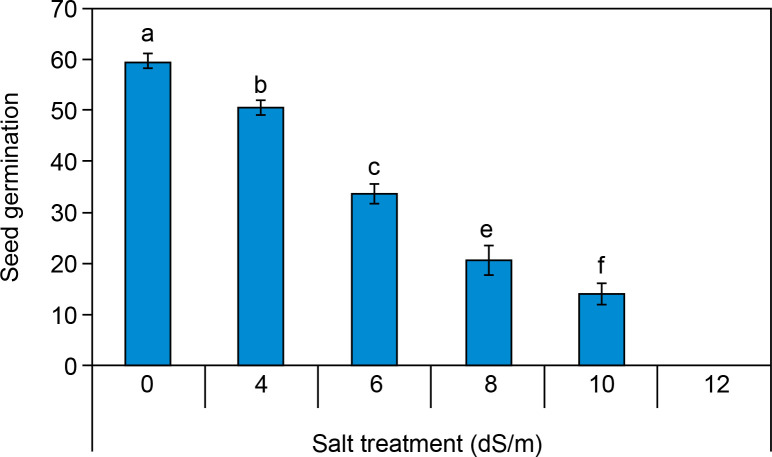
Effect of salt concentrations (0, 4, 6, 8, 10, and 12 dS/m) on seed germination percentage in *Spinacia oleracea* after 10 days of treatment under hydroponic conditions. (All experiments were performed in triplicate). Data is mean ± standard error (SE) scored and followed by a, b, c, d, e, and f letters indicating that there is a significant difference and level of significance at *p* ≤ 0.05 among treatments by Duncan’s multiple range test (DMRT)

The seed germination percentage of spinach seeds is illustrated in Figure 2, showing a significant variation between the control and salt-treated seeds. Under control conditions, seed germination was 59.6 ± 1.45%. Seed germination percentage was recorded and reduced percentage (14 ± 2% at 10 dS/m) with increasing concentration of salinity after 10 days. Seed germination was found to be inhibited at 12.0 dS/m salt concentration. There was no seed germination (0.0%) at all at a concentration of ECe 12.0 dS/m salt. Our findings align with the study conducted by Turhan et al. (2011), which examined the effects of different salt concentrations (0, 4.7, 9.4, and 14.1 dS/m) on seed germination in cabbage (*Brassica oleracea* capitata) and cauliflower (*Brassica oleracea* botrytis) species and it was found that an increasing salt concentration significantly decreases the percent seed germination. Several researchers also confirmed that salinity negatively affects seed germination in various crop species, including *Brassica* sp., *Oryza sativa*, *Posidonia*, *Triticum aestivum*, and *Zea mays* (Gul et al., 2013; Ibrahim et al., 2019). Seed germination percentage was considerably decreased in IR 20 and 29, IWA 11, NERICA1, 5, 12, 19, and POKKALI varieties of rice (*Oryza sativa*) due to increased salt stress. In the control, the seed germination percentage of these all varieties was greater than 90% under the control condition (0 dS/m salt concentration). Likewise, at the salinity level of 5.0 dS/m, a high germination rate (> 90%) was still observed in “NERICA 5” and “NERICA 12,” while “IR 20,” “IR 29,” and “NERICA 19” exhibited germination rates between 80% and 90% (Ologundudu et al., 2014). Similarly, Sneha et al. (2013) also investigated that as the salt concentration increases the rate of germination decreases in *Penicillium glaucum* too.

#### Measurement of root and shoot length

**Figure 3:**
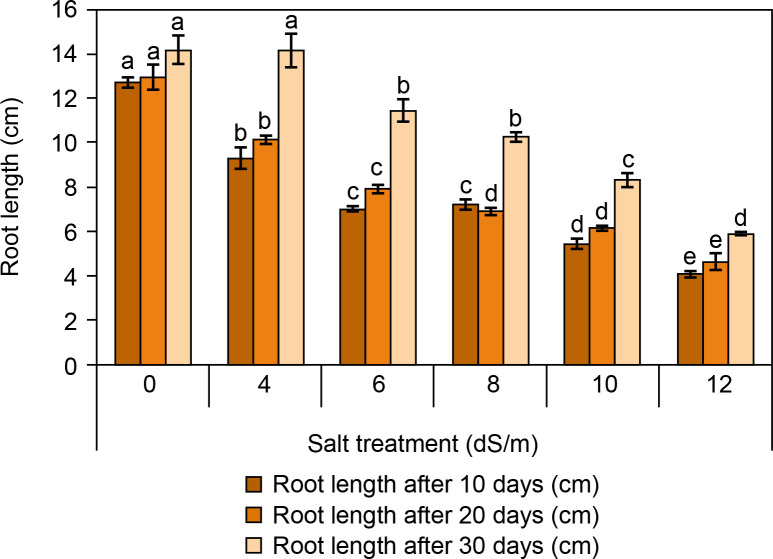
Influence of salt concentrations (0, 4, 6, 8, 10, and 12 dS/m) on root length in *Spinacia oleracea* was recorded after 10, 20, and 30 days of treatments under hydroponic conditions. Data is mean ± SE scored and followed by a, b, c, and d letters indicating that there is a significant difference and level of significance at *p* ≤ 0.05 among treatments by DMRT

**Figure 4:**
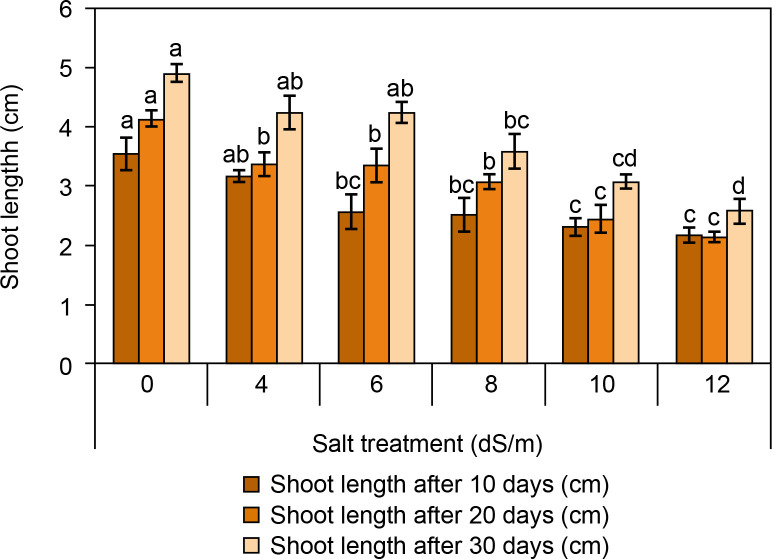
Influence of salt concentrations (0, 4, 6, 8, 10, and 12 dS/m) on shoot length in *Spinacia oleracea* was recorded after 10, 20, and 30 days of treatments under hydroponic conditions. Data is mean ± SE scored and followed by a, b, c, and d letters indicating that there is a significant difference and level of significance at *p* ≤ 0.05 among treatments by DMRT

The root and shoot lengths of spinach are presented in Figures 3 and 4. It was observed that the root and shoot length was found to be reduced with increasing salt concentration (Fig. 3). After 30 days, root and shoot length was highest in the control condition (root: 14.2 ± 0.5 cm and shoot: 4.9 ± 0.15 cm). At ECe 4.0 dS/m and 6 dS/m salt concentration, root and shoot length was observed better (root: 14.16 ± 0.19 cm and 11.4 ± 0.15 cm; shoot: 2.23 ± 0.28 cm, respectively) compared to ECe 8.0 dS/m and ECe 10.0 dS/m. The lowest root and shoot lengths were recorded at ECe 12.0 dS/m (root: 5.93 ± 0.35 cm; shoot: 2.57 ± 0.2 cm). Camlica and Yaldiz (2017) reported that salt stress negatively affects seedling growth. Similarly, a significant decline in seedling growth was observed in *Thymus maroccanus* (Belaqziz et al., 2009), as well as in basil, chamomile, and marjoram were also harshly declined depending on salt stress (Ali et al., 2007). Likewise, root and shoot length was significantly reduced from 38.83 cm in control to 9.44 cm at 200 mM and from 7.48 cm in control to 2.44 cm at 200 mM salt concentration respectively at 60 days of sowing (Kapoor and Pande, 2015).

#### Leaf relative water content

A decline in LRWC was observed in spinach leaves with increasing salt concentration (Table 1). LRWC was highest in the control plants compared to salttreated seedlings over 30 days. At ECe 4.0 dS/m (47.5 ± 0.43%), ECe 6.0 dS/m (47.1 ± 0.65%), and ECe 8.0 dS/m (45.3 ± 0.74%), the salt concentration embodies a good proportion of LRWC but is affected at ECe 10.0 dS/m (40.8 ± 0.51%) and ECe 12.0 dS/m (32.1 ± 0.86%) concentrations. When we compared the control spinach with 12.0 dS/m salt-treated spinach for RWC after 30 days, it was observed that there was about a 15% reduction in LRWC. Taffouo et al. (2017) reported similar effects of NaCl on relative water content in *Capsicum annuum* cultivars, with a significant decrease in RWC at 200 mM salinity in “Granada” and “Nobiliall” cultivars. Likewise, a negative effect of salt stress on RWC was observed at 120 mM NaCl in peanut (*Arachis hypogaea*) leaves of “Vanda,” “P244601,” and “Pl184948” varieties (Meguekam et al., 2021). Salt stress also reduced RWC in two wheat (*Triticum aestivum*) cultivars (Giza 168 and Gimeza 9), and among these two cultivars, the RWC reduction was found more pronounced in the “Giza 168” cultivar. Similarly, Kumar et al. (2021) also found that RWC dropped in both cultivars when NaCl concentration increased compared to the control, except 50 mm, where RWC increased to 7.99 and 6.06% in V11E0022 and V11E0135, respectively. Furthermore, a substantially larger drop in RWC was detected in V11E0135 than in V11E0022.

**Table 1:** Effect of salt concentrations (0, 4, 6, 8, 10, and 12 dS/m) on percent relative water content in shoots of *Spinacia oleracea* was recorded after 10, 20, and 30 days of treatments under hydroponic condition

Salt concentration (dS/m)	Relative water content (RWC) (%)		
	After 10 days	After 20 days	After 30 days
0	40.1 ± 0.66^a^	40.0 ± 0.12^a^	47.8 ± 1.00^a^
4.0	35.6 ± 0.34^b^	40.9 ± 0.77^a^	47.5 ± 0.43^a^
6.0	36.4 ± 0.54^b^	40.8±0.35^a^	47.1 ±0.65^a^
8.0	32.4 ± 0.50^c^	37.9 ± 0.48^b^	45.3 ± 0.74^a^
10.0	31.5 ± 0.74^c^	31.4 ± 0.59^c^	40.8 ± 0.51^b^
12.0	29.4 ± 0.69^d^	30.7 ± 0.54^c^	32.1 ±0.86^c^

Data is mean ± standard error (SE) scored and followed by a, b, c, and d letters are indicating that there is significant difference and level of significance at p ≤ 0.05 among treatments by Duncan’s multiple range test (DMRT).

#### Measurement of leaf area

Leaf surface area was also affected, with control leaves showing a surface area of 39.5 ± 0.69 cm^2^ after 30 days, while only 9 ± 0.12 cm^2^ surface area was seen at ECe 12 dS/m. Camlica and Yaldiz (2017) discussed that the morphological characteristics of plants were also affected negatively by exposure to salt-increasing concentration (Table 2).

**Table 2:** Effect of salt concentrations (0, 4, 6, 8, 10, and 12 dS/m) on leaf area in *Spinacia oleracea* was recorded after 10, 20, and 30 days of treatments under hydroponic condition

Salt concentration (dS/m)	Leaf area (cm^2^)		
	After 10 days	After 20 days	After 30 days
0	14.60 ± 0.26^a^	26.86 ± 0.19^a^	39.50 ± 0.69^a^
4.0	13.73 ± 0.43^ab^	19.96 ± 0.32^b^	25.03 ± 0.26^b^
6.0	13.26 0.15^b^	19.96 ± 0.26^b^	20.90 ± 0.49^c^
8.0	10.60 ± 0.31^c^	18.46 ± 0.26^c^	19.80 ± 0.15^c^
10.0	6.36 ± 0.23^d^	15.46 ± 0.38^d^	16.66 ± 0.24^d^
12.0	8.33 ± 0.44^e^	7.20 ± 0.25^e^	9.00 ± 0.12^e^

Data is mean ± SE scored and followed by a, b, c, d, and e letters are indicating that there is significant difference and level of significance at p ≤ 0.05 among treatments by DMRT.

Similarly, previous studies have shown that salinity stress affects the leaf area of several medicinal plants, including *Geranium* (Leithy et al., 2009), *Majorana hortensis* (Shalan et al., 2006), *Mentha pulegium* (Queslati et al., 2010), peppermint (Aziz et al., 2008), and *Thymus vulgaris* (Najafian et al., 2009). A significant difference was observed in both leaf tissue water content and leaf area between control and salt-treated plantlets.

### Biochemical parameters

#### Chlorophyll estimation

Chlorophyll content decreased with increasing salt concentration in *Spinacia oleracea* under hydroponic culture conditions (Table 3). Total chlorophyll content (Chl-a, and Chl-b) in leaves of spinach was significantly decreased with increased salt treatments than the control plantlets. Higher total chlorophyll content (4.0 dS/m — 3.54 ± 0.02 was observed in salt-treated plantlets when compared with controls (3.73 ± 0.02 mg/g) (Table 3). Increased NaCl concentration in the growth medium, Chl-a content was found to decrease by 52% in highyielding genotype Tema and by 57% in low-yielding genotype Djadida of *Phaseolus vulgaris* under high salt stress. In addition, Chl-b content was found to decrease by 33% in the high-yielding genotype Tema and by 43% in the lowyielding genotype Djadida of *Phaseolus vulgaris* under high salinity stress in comparison with controls. Thus, total chlorophyll content was recorded as reduced in both cultivars of *Phaseolus vulgaris* under high salt stress (Taibi et al., 2016). Yasar et al., (2008) have reported the influence of increasing NaCl levels on chlorophyll content in GS57 and *cv*. Fransiz 4F-89 varieties were analyzed after 7-days treatment to salt stress. In *cv. Fransiz 4F-89*, chlorophyll content gradually declined with increasing NaCl concentrations, while in the *GS57* genotype, chlorophyll content decreased at 50 mM NaCl concentration. Salinity levels had a significant effect on chlorophyll a, chlorophyll b, and total chlorophyll content. Increasing salinity from 0 to 6.0 dS/m led to a substantial reduction in total chlorophyll content. A similar decline in chlorophyll pigment levels was recorded in *Ocimum basilicum* plants exposed to 6.0 dS/m salinity stress (Heidari, 2012).

**Table 3:** Effect of salt concentrations (0, 4, 6, 8, 10, and 12 dS/m) on total chlorophyll content in leaf tissue of *Spinacia oleracea* grown under hydroponic condition

Salt concentration (dS/m)	Chlorophyll a (mg/g)	Chlorophyll b (mg/g)	Total chlorophyll (mg/g)
0	3.47 ± 0.16^a^	0.54 ± 0.02^e^	3.73 ± 0.02^a^
4.0	2.19 ± 0.02^b^	1.35 ± 0.01^a^	3.54 ± 0.02^b^
6.0	2.23 ± 0.02^b^	1.10 ± 0.01^c^	3.33 ± 0.01^c^
8.0	1.74 ± 0.03^c^	1.25 ± 0.01^b^	3.10 ± 0.06^d^
10.0	1.59 ± 0.03^cd^	0.75 ± 0.03^d^	2.47 ± 0.02^e^
12.0	1.44 ± 0.02^d^	0.54 ± 0.03^e^	1.95 ± 0.03^f^

Data is mean ± standard error (SE) scored and followed by a, b, c, d, e, and f letters indicating that there is a significant difference and level of significance at p ≤ 0.05 among treatments by DMRT.

#### Antioxidant enzyme assays

Antioxidant enzyme assays revealed that SOD, CAT, and APX activities in salt-treated spinach leaves increased with rising salt concentrations (Nikam et al., 2019; Bogoutdinova et al., 2020). Azooz et al. (2009) reported that plants activate robust antioxidant defense mechanisms, including SOD, CAT, and peroxidase, in response to salt stress.

**SOD** activity was found to be increased as compared to control with increasing salt concentration under 4.0, 6.0, 8.0, 10.0, and 12.0 dS/m NaCl treatments were monitored after 10, 20, and 30 days (Table 4). Salinity-induced increment in SOD activity (1.78 ± 0.03/g protein) was noted in leaves of spinach at 12.0 dS/m NaCl treatment after 30 days. It is also observed that higher SOD activity in leaves of spinach with increasing concentrations of salt and exhibited tolerance to salt stress. There is a significant difference in SOD activity recorded in spinach treated with 0.0 to 12 dS/m salt concentration. Zhang et al. (2013) reported that antioxidant enzyme activity increased in *Catharanthus roseus* cell suspension cultures under salinity stress. Similar findings were observed in common bean (*Phaseolus vulgaris*) (Jebara et al., 2005) and potato plants (Rahnama and Ebrahimzadeh, 2005) under salinity conditions. SOD is considered the most effective enzyme in scavenging reactive oxygen species (ROS), which cause oxidative stress induced by salinity (Taffouo et al., 2017). Taibi et al. (2016) reported that spinach treated with 12.0 dS/m NaCl exhibited 1.844/g protein SOD activity, which was higher than that of control plants. Bor et al. (2003) and Koca et al. (2006) further demonstrated increased SOD activity in wild beet species, validating a correlation between elevated SOD activity and salinity tolerance in different genotypes of wild beet.

**Table 4:** Influence of salt concentrations (0, 4, 6, 8, 10, and 12 dS/m) on superoxide dismutase activity in *Spinacia oleracea* after 10, 20, and 30 days of treatments under hydroponic conditions

Salt concentration (dS/m)	Superoxide dismutase activity (SOD) (content/g protein)		
	After 10 Days	After 20 Days	After 30 Days
0	0.73 ± 0.02^a^	1.13 ± 0.07^b^	1.48 ± 0.14^b^
4.0	0.71 ± 0.07^a^	1.42 ± 0.08^a^	1.74 ± 0.04^a^
6.0	0.74 ± 0.04^a^	1.36 ± 0.04^a^	1.73 ± 0.03^a^
8.0	0.79 ± 0.03^a^	1.38 ± 0.01^a^	1.81 ± 0.02^a^
10.0	0.81 ± 0.02^a^	1.48 ± 0.03^a^	1.83 ± 0.04^a^
12.0	0.81 ± 0.02^a^	1.43 ± 0.03^a^	1.78 ± 0.03^a^

Data is mean ± SE scored and followed by a, and b letters indicating that there is a significant difference and level of significance at p ≤ 0.05 among treatments by Duncan’s multiple range test DMRT.

**Figure 5:**
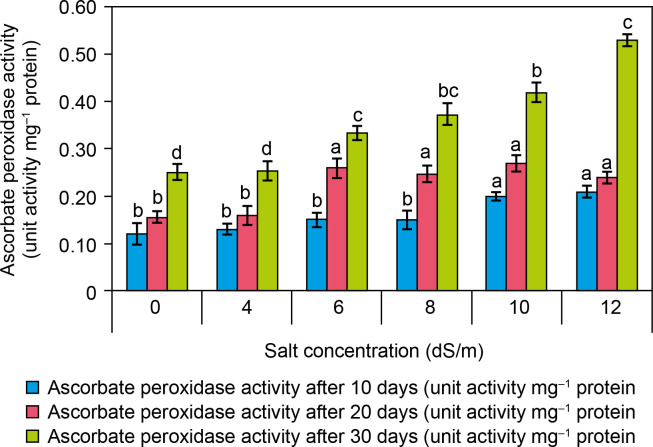
Effect of salt concentrations (0, 4, 6, 8, 10, and 12 dS/m) on Ascorbate peroxidase activity in plantlets of Spinacia oleracea after 10, 20, and 30 days of treatments under hydroponic conditions. Data is mean ± SE scored and followed by a, b, c, and d letters indicating that there is a significant difference and level of significance at *p* ≤ 0.05 among treatments by DMRT

**APX** activity (EC 1.11.1.11) was found to be increased as compared to control with increasing salt concentration (Fig. 5). At ECe 12.0 dS/m NaCl concentration, there was high antioxidant enzyme activity (0.53 ± 0.1/mg protein) was recorded as compared to control (0.25 ± 0.02/mg protein) after 30 days. There is a significant increase in APX activity recorded with increasing concentration of NaCl. At the physiological level, APX enzyme activity was found to increase in different plant species under saline environments. Likewise, at the molecular level, the APX gene modulation occurs in response to salinity stress (Caverzan et al., 2012). Taibi et al. (2016) reported that spinach treated with 12.0 dS/m NaCl exhibited 0.505 mg protein APX activity, which was higher than in control plants. Additionally, APX activity was significantly elevated in the high-yielding genotype “Tema” of *Phaseolus vulgaris* under a 200 mM NaCl environment.

**Figure 6:**
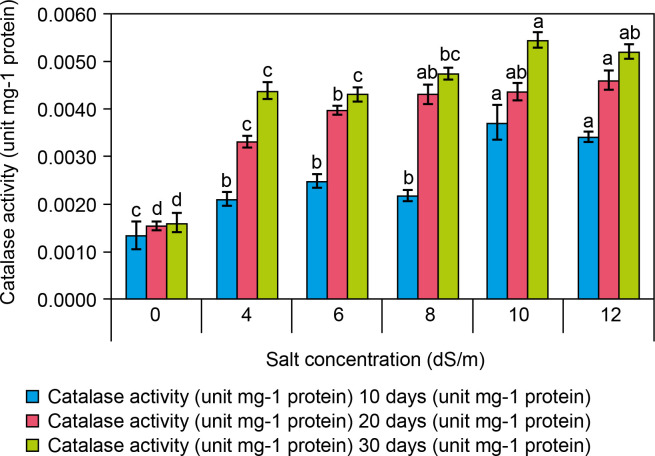
Effect of salt concentrations (0.0, 4, 6, 8, 10, and 12 dS/m) on catalase activity (unit/g protein) in plantlets of Spinacia oleracea after 10, 20, and 30 days of treatments under hydroponic conditions. Data is mean ± SE scored and followed by a, b, c, and d letters indicating that there is a significant difference and level of significance at *p* ≤ 0.05 among treatments by DMRT

**CAT** activity (EC 1.11.1.6) in spinach leaves significantly increased at 10 dS/m NaCl (0.0054 ± 0 mg protein) compared to the control (0.0016 ± 0 mg protein at 0 dS/m NaCl) after 30 days (Fig. 6). The following increases in CAT activity were recorded at different salt concentrations: 0.0044 ± 0 at 4 dS/m, 0.0043 ± 0 at 6 dS/m, 0.0047 ± 0 at 8 dS/m, 0.0054 ± 0 at 10 dS/m, and 0.0052 ± 0/mg protein at 12 dS/m salt) was recorded in leaves of salt-treated plantlets as compared to control plantlets. Therefore, the increased catalase activity in the leaves of salt-treated plantlets was not significantly correlated with the increasing salt concentration. Catalase is one of the most efficient enzymes in the scavenging ROS generated by salt-induced oxidative stress (Taffouo et al., 2017). Spinach treated at 12.0 dS/m NaCl exhibited 0.0055 mg protein CAT activity, which was higher than that of control plants under a saline environment (Taibi et al., 2016). CAT pathway is considered a useful process, and currently, CAT pathway is increasingly valued as a key component of photosynthesis and an essential part of salt stress responses in plants for avoiding ROS accumulation (Sofo et al., 2015).

### Proline content

**Figure 7:**
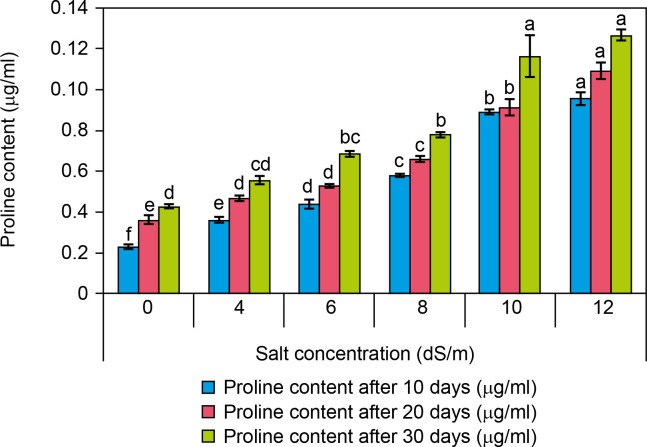
Influence of salt concentrations (0, 4, 6, 8, 10, and 12 dS/m) on proline content in plantlets of *Spinacia oleracea* after 10, 20, and 30 days of treatments under hydroponic conditions. Data is mean ± SE scored and followed by a, b, c, d, e, and f letters indicating that there is a significant difference and level of significance at *p* ≤ 0.05 among treatments by DMRT

Proline content was examined in control (0 dS/m NaCl) and salt-treated (4–12 dS/m NaCl) spinach plantlets revealing a significant difference between control and salt-exposed plants (Fig. 7). It was observed that at high salt concentration, the proline content was found to increase as compared to control presented (Fig. 5). The proline content was found to be increased as compared to the control with increasing salt concentration. Proline content in controls was found 0.024 ± 0 μg/ml while it was 0.055 ± 0 μg/ml at 4 dS/m, 0.068 ± 0 μg/ml at 6 dS/m, 0.077 ± 0 μg/ml at 8 dS/m, 0.11 ± 0.1 μg/ml at 10 dS/m, and 0.12 ± 0 μg/ml at 12 dS/m salt concentration treated plants after 30 days. A percent increase in proline content was recorded about 96% in salt-treated plantlets when compared to control plantlets. As per the obtained results, there was a significant increase in proline content in the salt-treated plantlets. Therefore, there was a significant difference recorded in proline content between control and salt-treated plantlets. Several researchers have shown that there is an accumulation of proline in saline-tolerant cultivars like *Cucumis melo* (Sarabi et al., 2017) and *Solanum tuberosum* (Jaarsma et al., 2013). Moreover, according to De la Torre-Gonzales et al. (2018) when individuals of *Lycopersicum esculentum* plant are grown under saline environments, the level of proline increase corresponded to enhancement in its tolerance to salinity. Here the results recorded are similar to our study like a rapid increase in proline content in leaves occurred when the plant was treated with 300 mM NaCl concentration (Hnilickova et al., 2021). Proline plays a protective role in salt-stressed plants by maintaining osmoregulation, neutralizing ROS, preserving cell membrane integrity, and stabilizing enzymes and proteins (Ashraf et al., 2007; Szabados and Savoure, 2010; Hnilickova et al., 2021). The enhancement in proline content after 21 days in two *Portulaca oleracea* genotypes (T-16 and WI-9) with 200 mM NaCl stress was investigated by Mulry et al. (2015). Yazici et al. (2007) recorded a 3-fold increase in proline content in *Portulaca oleracea* treated with 140 mM NaCl for 30 days. Leal et al. (2020) found that in *Spinacia oleracea* L. cv. “Viroflay,” salinity enhanced osmotic adjustment and leaf succulence while reducing leaf water potential. The positive responses to salinity were supported by increased salt content in leaves and fresh weight, with hydroponic conditions yielding optimal results. They suggested the use of plastic covers or hydroponic techniques for spinach growing, particularly when irrigating with brackish water.

### Protein content

**Figure 8:**
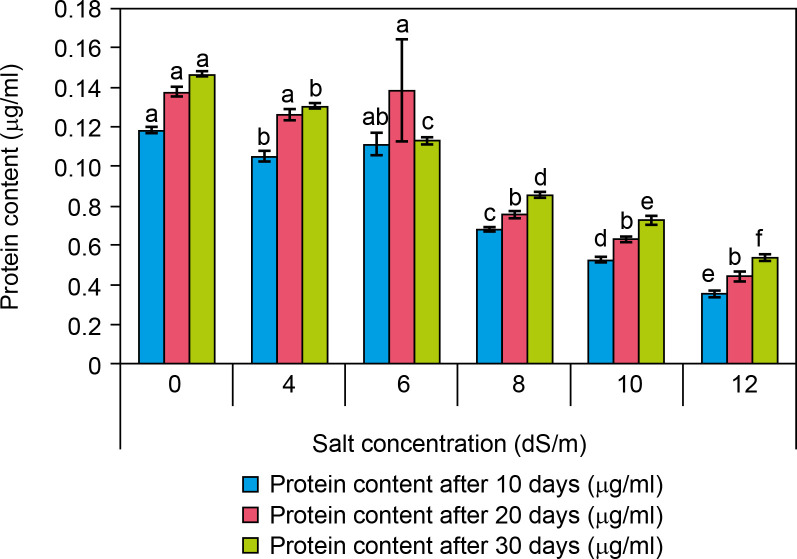
Effect of salt concentrations (0, 4, 6, 8, 10, and 12 dS/m) on protein content in plantlets of Spinacia oleracea after 10, 20, and 30 days of treatments under hydroponic conditions. Data is mean ± SE scored and followed by a, b, c, d, e, and f letters indicating that there is a significant difference and level of significance at *p* ≤ 0.05 among treatments by DMRT

Among the biochemical parameters, it was observed that a high salt concentration significantly decreased protein content after 30 days and it was presented as Figure 8. The protein content was significantly decreased (0.054 ± 0 μg/ml at 12 dS/m) in salt-treated plantlets of spinach as compared to control (0.14 ± 0 μg/ml) after 30 days of interval. The protein content was found to decrease with increasing salt concentrations like 0.13 ± 0 at 4 dS/m, 0.11 ± 0 at 6 dS/m, 0.08 at 8 dS/m, and 0.073 μg/ml at 10 dS/m.

**Figure 9:**
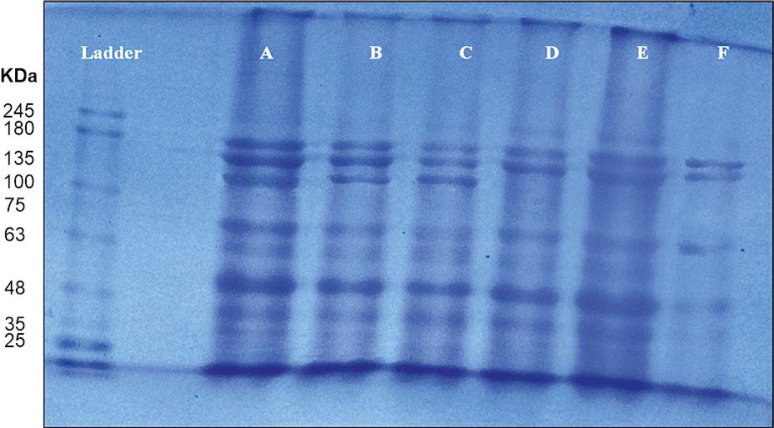
Total protein content extracted from leaves of salt treated spinach and proteins separation was performed by using SDS-PAGE gel electrophoresis. The different protein bands (size of 25, 35, 63, 75, 100, 135, 180, and 245 kDa) can be observed in the salt treated spinach leaf samples. A pre-stained protein ladder was used as known molecular mass marker (Ladder lane). Pattern of protein distribution in the leaves of spinach studied, where A is ECe 0.0 dS/m, B is 4.0 dS/m, C is 6.0 dS/m, D is 8.0 dS/m, E is 10 dS/m, and F is 12 dS/m salt concentrations

Protein expression in control and salt-treated plantlets was further examined using SDS-PAGE analysis, as shown in Figure 9. A total of nine protein bands appeared across six treated samples and the control (ECe 0.0 dS/m) were grouped as three distinct protein bands (D, E, and F). These SDS protein bands belong to distinct molecular weights ranging from 25 to 245 kDa. The additional protein bands (135 kDa protein) were observed at control 0.0 dS/m and treated ECe 04 and 06 dS/m salt concentrations in spinach leaves, however, this particular protein band disappeared at ECe 08, 10, and 12 dS/m salt concentrations. Likewise, SDS-PAGE analysis of total proteins extracted from leaves of control and hydroponically NaCl-treated plantlets of *Broussonetia papyrifera* revealed that in the leaves increased salt (100 and 150 mM NaCl) stress was associated with a decrease or disappearance of some protein bands (Zhang et al., 2013). Similarly, the highest leaf protein content in control and decreased concentration of leaf protein was recorded in 200 mM salinity level exposed plants of *Portulaca oleracea* (Parvaneh et al., 2012). Parvaiz and Satyavati, (2008) and Doganlar et al. (2010) also reported reduced total protein content in *Amaranthus tricolor*, *Brugiera parviflora*, *Lycopersicon esculentum*, *Oryza sativa*, and *Vicia faba* under 200 mM NaCl stress. However, in *Lupinus angustifolius*, total protein content remained unaffected by salt stress, whereas a decline was recorded in the root, young leaves, and old leaves of *Helianthus annuus* and *Coleus blumei* (Doganlar et al., 2010).

The results of the present study indicate that protein biosynthesis decreases with increasing salt concentrations.

## Conclusions

This study investigated the impact of salt concentrations on *Spinacia oleracea* seedlings under a hydroponic culture system, revealing significant effects on various morphological and biochemical parameters. Morphological traits, biochemical markers, and antioxidant enzyme activity were all influenced by increasing salinity levels. As the salt concentration increased from ECe 4.0 to ECe 12.0 dS/m, various physiological and biochemical processes were directly affected under hydroponic conditions. The results demonstrated a notable reduction in seed germination percentage, root and shoot length, LRWC, leaf area, and protein content with increasing salinity. SDS-PAGE analysis further confirmed a decline in protein content through changes in protein band patterns. Therefore, the spinach (*S. oleracea*) grown under a hydroponic culture system can tolerate salt stress up to ECe 6.0 dS/m and more increased (ECe 8.0, 10, and 12 dS/m) salt concentration that adversely affects overall growth and biochemical performance. The present study opens new corridors as the vegetable crops could be grown under saline water using a hydroponic culture system instead of soil, this study could be a valuable addition to the crop spinach.
